# Proceedings of the International Workshop ‘Integration of International Expertise in the Development of a Mental Health Surveillance System in Germany’: Berlin, Germany. 28-29 November 2019

**DOI:** 10.1186/s12919-020-00186-0

**Published:** 2020-05-04

## Introduction

### I1 Towards the development of a National Mental Health Surveillance System in Germany

#### Julia Thom, Diana Peitz, Christina Kersjes, Heike Hölling, Elvira Mauz

##### Department of Epidemiology and Health Monitoring, Robert Koch Institute, Berlin, Germany

###### **Correspondence:** Elvira Mauz (MauzE@rki.de)

In 2019, the Robert Koch Institute (RKI) as the national Public Health Institute in Germany was commissioned by the Federal Ministry of Health to develop a concept for continuous health reporting on mental health in Germany. Meaningful data is required since mental health has strong public health relevance due to high prevalence and burden of psychological distress and mental disorders and the improvable care situation. Furthermore there is still unexploited potential to promote positive mental health. In Germany, almost one in three adults (27.8%) fulfils the criteria of a mental disorder within one year [1, 2]; the prevalence of emotional and behavioral disorders in children and adolescents is estimated at 10 to 20% [3]. Depressive disorder ranks globally as well as nationally among the most significant causes of Years Lost due to Disability (YLDs) [4, 5]. Additionally physical and mental health is closely interwoven: mental disorders deteriorate the course of somatic illnesses and vice versa, somatic illnesses represent a risk factor for the emergence of mental disorders. Against this background, the WHO has included mental disorders in its list of central non-communicable diseases [6]. Moreover, the establishment of Mental Health Information Systems is one of the four priority objectives of the WHO's Mental Health Action Plan. Besides mental disorders, this action plan targets on mental well-being as an integral component of health in general [7].

In order to face this task, a German indicator-based Mental Health Surveillance System (MHS) is under development. The proceeding comprises a consensus process among public mental health experts and stakeholders. Together with experts from Canada and Europe, representatives of 21 national and international institutions were invited to the workshop ‘Integration of International Expertise in the Development of a Mental Health Surveillance System in Germany’, held in Berlin, Germany on November 28-29, 2019. Main goals were (a) to share experience on existing mental health surveillance systems, (b) to present objective and methods of the German approach to the national expert committee, (c) to strengthen international collaboration and (d) to discuss central challenges and controversial issues.

The introduction into the development of a national MHS at the Robert Koch Institute (S1) was given by Elvira Mauz (RKI, Berlin) by outlining the project’s background and workflow. Daniel Chisholm (WHO Europe, Copenhagen) reported on indicators development for the WHO Mental Health Atlas in the context of Mental Health Action Goals and Sustainable Development Goals (S2). Emily Hewlett (OECD, Paris) focused on the international comparability of mental health indicators (S3), whereas Daniela Schuler (Swiss Health Observatory, Neuchâtel) presented the national MHS in Switzerland (S4). Wolfgang Gaebel (LVR-Klinikum, Düsseldorf) presented indicators for mental healthcare quality which where developed for international comparison for the Danube Region (S5). Heather Orpana (Public Health Agency Canada, Ottawa) gave an overview of MHS Systems in Canada with a special focus on Positive Mental Health (S6). In two discussion sessions the following questions were addressed of (a) how a balance of indicators between mental health promotion, prevention and care of mental disorders might be achieved and (2) which mental disorders should be the focus within a German MHS.

**References**

1. Jacobi F, Höfler M, Siegert J, Mack S, Gerschler A, Scholl L, Busch MA, Hapke U, Maske U, Seiffert I *et al*: **Twelve-month prevalence, comorbidity and correlates of mental disorders in Germany: the Mental Health Module of the German Health Interview and Examination Survey for Adults (DEGS1-MH)**. *International Journal of Methods in Psychiatric Research* 2014, **23**(3):304-319.

2. Jacobi F, Höfler M, Strehle J, Mack S, Gerschler A, Scholl L, Busch MA, Hapke U, Maske U, Seiffert I *et al*: **Twelve-months prevalence of mental disorders in the German Health Interview and Examination Survey for Adults - Mental Health Module (DEGS1-MH): a methodological addendum and correction**. *International Journal of Methods in Psychiatric Research* 2015, **24**(4):305-313.

3. Barkmann C, Schulte-Markwort M: **Prevalence of emotional and behavioural disorders in German children and adolescents: a meta-analysis**. *J Epidemiol Community Health* 2012, **66**(3):194-203.

4. Kyu HH, Abate D, Abate KH, Abay SM, Abbafati C, Abbasi N, Abbastabar H, Abd-Allah F, Abdela J, Abdelalim A *et al*: **Global, regional, and national disability-adjusted life-years (DALYs) for 359 diseases and injuries and healthy life expectancy (HALE) for 195 countries and territories, 1990–2017: a systematic analysis for the Global Burden of Disease Study 2017**. *The Lancet* 2018, **392**(10159):1859-1922.

5. Plass D, Vos T, Hornberg C, Scheidt-Nave C, Zeeb H, Krämer A: **Trends in disease burden in Germany: results, implications and limitations of the Global Burden of Disease study**. *Dtsch Arztebl Int* 2014, **111**(38):629-638.

6. Nishtar S, Niinistö S, Sirisena M, Vázquez T, Skvortsova V, Rubinstein A, Mogae FG, Mattila P, Ghazizadeh Hashemi SH, Kariuki S *et al*: **Time to deliver: report of the WHO Independent High-Level Commission on NCDs**. *The Lancet* 2018.

7. World Health Organization: **Mental health action plan 2013-2020**. In*.* Geneva, Switzerland; 2013.

## Speakers Presentations

### S1 Mental Health Surveillance in Germany – Status quo and Perspectives

#### Elvira Mauz, Christina Kersjes, Diana Peitz, Heike Hölling, Julia Thom

##### Department of Epidemiology and Health Monitoring, Robert Koch Institute, Berlin, Germany

###### **Correspondence:** Elvira Mauz (MauzE@rki.de)

Since 2008, the Robert Koch Institute (RKI) has established a health monitoring of non-communicable diseases for Germany [1]. An indicator based systematic NCD-Surveillance is being developed since 2015, focusing first of all on diabetes [2]. According to the priority objectives of the WHO's Mental Health Action Plan [3], a Mental Health Surveillance (MHS) should be an integral part. The main aim of a MHS is to report selected core mental health indicators and their changes over time on a regular basis. To do so, all relevant data from different sources should be integrated. The systematic and continuous quantification of these indicators should create a reliable database for evidence-based policy advice and accompanying research on public health measures. This should contribute to protect and promote mental health in the population.

In March 2019, the Federal Ministry of Health, funded a three year pilot phase with the main aims of (a) developing an action-guiding framework and a core set of indicators for public mental health, (b) checking the relevant data sources and data gaps; (c) first quantification of core indicators based on available primary and secondary data and (d) conception and testing of an extended Mental Health Survey Inventory to close existent data gaps. Subsequently, the core mental health indicators, the continuous data collection within the health monitoring system of the RKI [1] and the result communication should be integrated into a broader NCD surveillance.

In the first project period, a comprehensive international scoping review was conducted to identify possible indicators of public mental health. This resulted in a set of 184 preliminary indicators. These indicators were assigned to a preliminary framework model “Promoting and Maintaining Public Mental Health”. This framework was developed in a focus group of experts and currently comprises five action goals: (1) “Promoting the mental well-being of all people”, (2) “Reducing the risks of mental disorders”, (3) “Improving mental health care”, (4) “Reducing the burden of disease & enabling participation” and (5) “Increasing knowledge and acceptance”.

In the following project period, feasible and central indicators that can cover this comprehensive range of issues in a meaningful and internationally comparable manner are to be identified. To achieve this, the prioritization and reduction of measures and the definition of a final framework model are conducted in consensus with international and national experts from science, health care practice, patient representation and health policy. The consensus process follows the course of a structured multi-stage Delphi procedure until mid-2020 with this workshop as a kick-off meeting, followed by two online surveys and another workshop.

**References**

1. Kurth BM, Lange C, Kamtsiuris P, Hölling H: **Health monitoring at the Robert Koch Institute. Status and perspectives**. *Bundesgesundheitsblatt, Gesundheitsforschung, Gesundheitsschutz* 2009, **52**(5):557-570.

2. Gabrys L, Heidemann C, Schmidt C, Baumert J, Teti A, Du Y, Paprott R, Ziese T, Banzer W, Böhme M *et al*: **Selecting and defining indicators for diabetes surveillance in Germany**. In: *Journal of Health Monitoring.* vol. 3; 2018: 3-21.

3. World Health Organization: **Mental health action plan 2013-2020**. In*.* Geneva, Switzerland; 2013.

### S2 WHO’s Mental Health Atlas and other core Indicators for the SDG era

#### Dan Chisholm, Lei B Jobe

##### Mental health Programme, Division of Noncommunicable Diseases and Health Promotion through the Life-course, WHO Regional Office for Europe, Copenhagen, Denmark

###### **Correspondence:** Dan Chisholm (chisholmd@who.int)

In 2001, the World Health Organization (WHO) initiated its Mental Health Atlas project to address the lack of data collection on mental health indicators. Since then, updates of the Atlas have been produced in 2005, 2011, 2014 and 2017 [1], with the last two iterations assuming new importance as the collected data are used to inform on progress towards the objective and targets of the *Comprehensive Mental Health Action Plan 2013-2020* (now extended to 2030).

**Fig. 1 (abstract S2).**
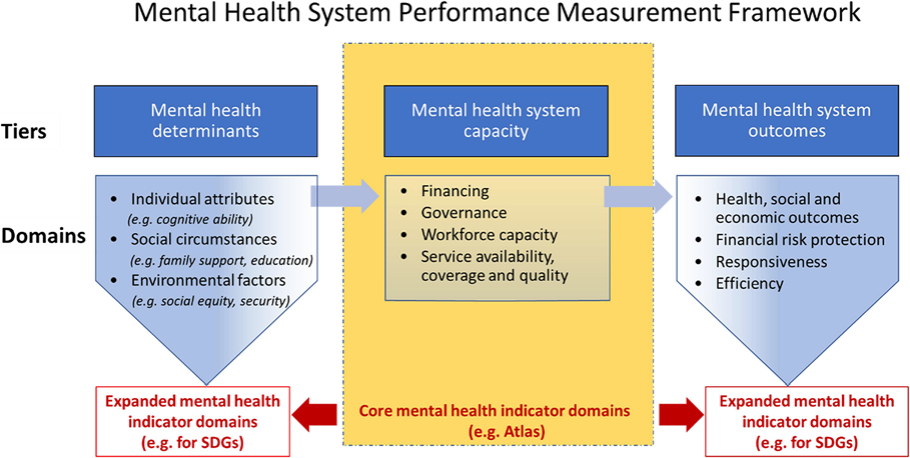
Mental Health System Performance Measurement Framework

To date, the Mental Health Atlas has mainly focused on the collection of data for indicators related to the capacity of health systems to respond to the public health burden of mental health conditions, such as the availability of policies, laws, resources and services. As governments, WHO and other international partners work towards the realization of new global goals for universal health coverage and sustainable development [2], mental health system performance assessment needs to expand beyond core features of “*mental health system capacity*” to the inter-related tiers of “*mental health determinants*”, and “mental *health system outcomes*” (Figure 1). Collecting data related to the *determinants of mental health* will assist decision-makers to better appreciate the underlying factors behind high and increasing levels of mental health need in the population, while assessment of *health systems outcomes* will provide decision-makers with better information on the impact and quality of mental health services. Each tier has domains which can be monitored by collecting a small number of carefully selected indicators that are meaningful and appropriate to health system planners and stakeholders.

WHO’s Mental Health Atlas already serves as an essential mechanism for monitoring core aspects of mental health system functioning over time and between countries. However, new efforts are now needed to better understand and measure the antecedents to and consequences of these mental health system activities, which will also contribute towards implementing and monitoring the SDGs.

**References**

1. World Health Organisation: **Mental Health Atlas 2017**. Geneva: WHO; 2018.

2. Patel V, Saxena S, Lund C, Thornicroft G, Baingana F, Bolton P, Chisholm D, Collins PY, Cooper JL, Eaton J *et al*: **The Lancet Commission on global mental health and sustainable development**. *The Lancet* 2018, **392**(10157):1553-1598.

### S3 OECD Development of Internationally Comparable Mental Health Indicators

#### Emily Hewlett

##### OECD, Paris, France

###### **Correspondence:** Emily Hewlett (Emily.HEWLETT@oecd.org)

The OECD helps countries achieve high-performing health systems by measuring health system inputs, outcomes, and resource use, and analyses policies that improve access, efficiency, and quality of health care. Given the significant burden of mental ill-health, for individuals, societies and economies, there is considerable interest in ways to strengthen mental health systems, and measure performance in an objective and standardised way.

Indicators related to mental health or mental health care included in the OECD Health Statistics 2019 (OECD, 2019) include number of psychiatric beds, average length of stay in a psychiatric beds, number of psychiatrists. OECD Health Statistics 2019 also includes five indicators in the area of quality of care and outcomes related to mental health: in-patient suicides among people diagnosed with a mental disorder, deaths after discharge from suicide among people diagnosed with a mental disorder, and excess mortality for patients with schizophrenia, bipolar disorder, or severe mental illness. The latter indicators were developed under the OECD’s Health Care Quality and Outcomes (HCQO) project; through the use of a structured review process, expert panels evaluated and recommended indicators related to quality of mental health care for further consideration in 2004 and 2012 (Hermann et al., 2004, Hewlett and Moran, 2014).

The OECD is developing new internationally comparable mental health indicators. Firstly, the OECD Mental Health Performance Benchmarking project will identify and collect relevant indicators – some qualitative, some quantitative – to assess and compare mental health performance. Secondly, under the Patient-Reported Indicators Surveys (PaRIS), a working group of OECD member country representatives are working to identify ways that mental health patient-reported outcome measures (PROM) and experience measures (PREM) could be collected and reported in an internationally comparable way. This work seeks to accelerate the adoption and reporting of validated, standardised, internationally comparable patient-reported indicators, and bring new insights into the impact of mental health care on service users’ lives (OECD, 2019). In addition, the PaRIS Survey of Patients with Chronic Conditions, which will garner first results from mid-2021, will be the first international survey of patient-reported health outcomes and experiences of adults with one or more chronic conditions who are treated in primary or ambulatory health care settings, and will cover some chronic mental disorders (OECD, 2019).

**References**

1. OECD. OECD Health Statistics 2019. *From:**https://stats.oecd.org/*

2. Hermann, R, Mattke S, and the Members of the OECD Mental Health Care Panel. Selecting indicators for the quality of mental health care at the health systems level in OECD countries. OECD Health Technical Papers No. 17. 2004. OECD Publishing, Paris. *Retrieved on 12 November 2019 from:**http://www.oecd.org/els/health-systems/33865630.pdf*

3. Hewlett E, Moran V. Making Mental Health Count: The Social and Economic Costs of Neglecting Mental Health Care. OECD Health Policy Studies. 2014. OECD Publishing, Paris. Retrieved *on 12 November 2019 from:*10.1787/9789264208445-en.

4. OECD. Measuring What Matters: the Patient-Reported Indicator Surveys. Patient-reported indicators for assessing health system performance. 2019. *Retrieved on 12 November 2019 from:*https://www.oecd.org/health/health-systems/Measuring-what-matters-the-Patient-Reported-Indicator-Surveys.pdf

5. OECD, Putting people at the centre of ehalth care. PaRIS survey of Patients with Chronic Conditions. 2019. *Retrieved on 12 November 2019 from:*https://www.oecd.org/health/health-systems/PaRIS-survey-Patients-with-Chronic-Conditions-June-2019.pdf

### S4 Mental Health Surveillance in Switzerland

#### Daniela Schuler

##### Swiss Health Observatory (Obsan), Neuchâtel, Switzerland

###### **Correspondence:** Daniela Schuler (Daniela.Schuler@bfs.admin.ch)

In Switzerland, mental health is monitored by the Swiss Health Observatory (Obsan). It is funded by the Confederation as well as the Swiss cantons and its main task consists of analysing available health related data in Switzerland to provide support for the Confederation, the cantons and health service institutions. Obsan does not conduct statistical surveys itself, but concentrates on using existing data. It therefore relies on various data sources in order to monitor mental health and mental health care in Switzerland. The most important data sources are the Swiss Health Survey and the Hospital Medical Statistics. The Swiss Health Survey is conducted every five years and surveys a representative, national population sample. It includes questions concerning health status, health related behavior and health care utilisation. The Hospital Medical Statistics is a national registry of all inpatient cases/patients in Swiss hospitals. It contains, for example, information on diagnoses and treatments. Other important data sources are the national Causes of Death Statistics and (disability) insurance data.

Although Obsan has access to a large variety of existing data, only a fragmented picture of the mental health situation in Switzerland can be drawn. Main reason is that the surveys and registries were originally not designed with the purpose of mental health surveillance. There is no national concept for the collection of mental health data in Switzerland.

Mental health monitoring by Obsan consists mainly of two efforts. First, several specific indicators on mental health are made publicly available on the Obsan website (www.obsan.ch) [1]. The indicators are interactive and present results at the sociodemographic and regional level and show the temporal evolvement, as well. Second, the Obsan publishes a comprehensive report on mental health in Switzerland every four to five years, including various topics such as the general mental health of the population, protective and risk factors, the use of medical services, occurrence of suicides, and the costs of mental illness [2, 3]. In addition to the report, there are further (smaller) publications (e.g. fact sheets) that focus on specific topics around mental health issues in Switzerland [e.g. 4, 5, 6].

**References**

1. **Mental Health Indicators** [https://www.obsan.admin.ch/de/indicators/search?keys=Psychische+Gesundheit]

2. Schuler D., Burla L.: **Psychische Gesundheit in der Schweiz. Monitoring 2012**. In: *Obsan Bericht 52.* Neuchâtel: Schweizerisches Gesundheitsobservatorium; 2012.

3. Schuler D., Tuch A., Buscher N., Camenzind P.: **Psychische Gesundheit in der Schweiz. Monitoring 2016**. In: *Obsan Bericht 72.* Neuchâtel: Schweizerisches Gesundheitsobservatorium; 2016.

4. Peter C., Tuch A.: **Suizidgedanken und Suizidversuche in der Schweizer Bevölkerung**. In: *Obsan Bulletin.* vol. 7/2019. Neuchâtel: Schweizerisches Gesundheitsobservatorium; 2019.

5. Schuler D., Tuch A.: **Fürsorgerische Unterbringungen in Schweizer Psychiatrien**. In: *Obsan Bulletin.* vol. 2/2018. Neuchâtel: Schweizerisches Gesundheitsobservatorium; 2018.

6. Schuler D., Tuch A., Peter C.: **Psychische Gesundheit. Kennzahlen 2017**. In: *Obsan Bulleting.* vol. 5/2018. Neuchâtel: Schweizerisches Gesundheitsobservatorium; 2019.

### S5 Mental Healthcare Surveillance in the Danube Region

#### Gaebel W^1,2^, Kerst A^1,2^, Stricker J^1,2^, Onus F^1^, Chisholm D^3^, Hinkov H^4^, Höschl C^5^, Kapocs G^6,11^, Kurimay T^6,12^, Lecic Tosevski D^7^, Milosavljevic M^8^, Nakov V^9^, Winkler P^10^, Zielasek J^13^, Lehmann I^13^

##### ^1^LVR-Klinikum Düsseldorf, Department of Psychiatry and Psychotherapy, Medical Faculty, Heinrich-Heine-University, Düsseldorf, Germany; ²WHO Collaborating Centre on Quality Assurance and Empowerment in Mental Health, Düsseldorf, Germany; ^3^WHO Regional Office for Europe, Copenhagen, Denmark; ^4^NCPHA-National Center of Public Health and Analyses, Sofia, Bulgaria; ^5^National Institute of Mental Health, Klecany, Czech Republic; ^6^Buda Family Centred Mental Health Centre, Department of Psychiatry and Psychiatric Rehabilitation, Teaching Department of Semmelweis University, Saint John Hospital, Budapest, Hungary; ^7^Serbian Academy of Sciences and Arts, Belgrade, Serbia; ^8^Institute of Mental Health, School of Medicine, University of Belgrade, Serbia; ^9^Mental Health, National Center of Mental Health and Analyses, Sofia, Bulgaria; ^10^Department of Social Psychiatry, National Institute of Mental Health, Klecany, Czech Republic; ^11^Institute for Behavioral Sciences, Semmelweis University, Budapest, Hungary; ^12^Department of Psychiatry and Psychotherapy, Semmelweis University, Budapest, Hungary; ^13^LVR-Institute for Healthcare Research, Cologne, Germany

###### **Correspondence:** Wolfgang Gaebel (wolfgang.gaebel@uni-duesseldorf.de)

Quality indicators (QI) are quantitative measures to monitor and evaluate the quality of structures, processes and outcomes of mental healthcare [1]. To date, various quality indicators are available for national, regional and international use [2,3,4,5,6]. However, for countries of the Danube region, quality indicators for mental health surveillance are still missing. The aim of the present project (Development and Implementation of Quality Indicators for Mental Healthcare in the Danube Region; DAQUMECA) conducted by the LVR-Institute for Healthcare Research (Germany) together with a country consortium (Bulgaria, Czech Republic, Hungary, Serbia) and the WHO Regional Office for Europe was (1) to systematically develop a set of quality indicators for the involved countries and (2) to run a pilot feasibility study of data collection for these quality indicators [7,8]. Based on a systematic literature review, we selected a set of QI (n=26). Subsequently, the selected indicators were rated in a two-stage Delphi study regarding their estimated relevance, validity and availability. The Delphi panel included relevant stakeholders (n=18) from the four involved Danube countries. Twenty-one QI were included in the final set of indicators to be tested in a pilot feasibility study. We collected data from different data sources retrospectively for 2017 and 2018 by means of the best available, most standardized, trustworthy, and up-to-date data in each country. In the Delphi study, the panelists rated the relevance of the selected QI as higher than their validity. There were no substantial country differences in these ratings. The expected data availability, however, differed strongly among QI (ranging from 6% to 94%). In the pilot feasibility study, data were available for 18/21 QI in Hungary, 17/21 QI in Bulgaria, 17/21 QI in the Czech Republic and 8/21 QI in Serbia. In sum, there was consensus among mental healthcare experts regarding the relevance and validity of the proposed QI. The lower ranking of validity compared to relevance corresponds to the scattered data availability and impeded accessibility reflected in the pilot feasibility study. Despite great interest and openness to assess and monitor the status and effects of mental healthcare reform processes, results are demonstrating the need for further efforts towards a more comprehensive, dynamic, and IT-based routine monitoring for assessing, planning and reforming national mental healthcare quality. We are currently planning a follow-up project aiming to further support reform processes by developing and implementing a transnational digital platform.

**References**

1. Donabedian A: The quality of care. How can it be assessed? JAMA 1988, 260:1743-1748.

2. Fisher CE, Spaeth-Rublee B, Pincus HA: For the IIMHL Clinical Leaders Group: Developing mental health-care quality indicators: toward a common framework. Int J Qual Health C 2013, 25:75-80.

3. Gaebel W, Becker T, Janssen B, Munk-Jorgensen P, Musalek M, Rössler W, Sommerlad K, Tansella M, Thornicroft G, Zielasek J: European Psychiatric Association (EPA) guidance on the quality of mental health services. Eur Psychiatry 2012, 27(Suppl 2):87–113.

4. Gaebel W, Großimlinghaus I, Heun R, Janssen B, Johnson B, Kurimay T, Montellano P, Muijen M, Munk-Joergensen P, Rössler W, Ruggeri M, Thornicroft G, Zielasek J: European Psychiatric Association (EPA) guidance on quality assurance in mental healthcare. Eur Psychiatry 2015, 30:360-387.

5. Großimlinghaus I, Falkai P, Gaebel W, Janssen B, Reich-Erkelenz D, Wobrock T, Zielasek J: Developmental process of DGPPN quality indicators. Nervenarzt 2013, 84:350-365.

6. Hermann R, Mattke S: Selecting indicators for the quality of mental health care at the health systems level in OECD Countries (OECD Health Technical Papers No. 17). Paris: OECD Publishing; 2004.

7. Lehmann I, Chisholm D, Hinkov H, Höschl G, Kapócs G, Kurimay T, Lecic-Toseviski D, Nakov V, Réthelyi JM, Winkler P, Zielasek J, Gaebel W: Development of quality indicators for mental healthcare in the Danube region. Psychiatria Danubina 2018, 30(Suppl. 2):197-206.

8. Gaebel W, Lehmann I, Chisholm D, Hinkov H, Höschl C, Kapócs G, Kurimay T, Lecic Tosevski D, Nakov V, Winkler P, Zielasek J: Quality indicators for mental healthcare in the Danube Region – Results from a pilot feasibility study. Manuscript submitted for publication.

### S6 Mental health surveillance at the Public Health Agency of Canada

#### Heather M Orpana PhD ^1,2^, Melanie Varin MSc^1^, Vera Grywacheski MPH^1^

##### ^1^Centre for Surveillance and Applied Research, Public Health Agency of Canada, Ottawa, Canada; ^2^School of Epidemiology and Public Health, University of Ottawa, Ottawa, Canada

###### **Correspondence:** Heather M Orpana (heather.orpana@canada.ca)

The Public Health Agency of Canada (PHAC) has a mandate to prevent disease and injuries and promote good physical and mental health. As part of its activities, PHAC develops and implements national surveillance systems, including those focused on mental illness, positive mental health, suicide, and substance-related harms.

Beginning in 2014, PHAC began the development of the Positive Mental Health Surveillance Indicator Framework (1) to fill an identified gap – data about positive mental health from a strengths-based perspective. It includes positive mental health outcomes, and risk and protective factors from a socioecological perspective. Data are primarily from ongoing surveys, such as the Canadian Community Health Survey (CCHS) for youth and adults.

Beginning in 2015, PHAC developed the Suicide Surveillance Indicator Framework (2), which includes suicide and self-inflicted injury outcomes, as well as risk and protective factors. Data are from vital statistics, administrative sources, and surveys.

Mental illness surveillance by PHAC relies primarily on two sources. Self-reported diagnosis of mood and/or anxiety disorders is included on a regular basis in the CCHS annual cycles. The Canadian Chronic Disease Surveillance System (3) is used to estimate the use of health care services for mental illness in general and for mood and anxiety disorders, and schizophrenia specifically using health care administrative data. Statistics Canada has implemented two CCHS focus surveys on mental health (2002, 2014) (4), which included implementing a modified WHO Composite International Diagnostic Interview for a limited number of disorders.

These existing systems and activities provide an overview of the state of mental health in Canada; however, gaps have been identified in the area of substance-related harms, particularly in the context of a steep rise in drug poisoning deaths (5). Initial surveillance activities in the area of substance-related harms focused on timely data on apparent opioid-related deaths. More recently, PHAC has broadened these activities to include a range of substance-related harms, and is now developing a framework for the public health surveillance of substance-related harms.

An overarching theme to these activities is the inter-relatedness between positive mental health, mental illness, suicide, and substance-related harms. In many cases, an outcome in one framework is a risk or protective factor in another framework.

Surveillance data are essential to identifying the scope of a health issue, understanding its distribution, and monitoring changes over time. Surveillance of mental health and associated concepts remains a priority for PHAC.

**References**

1) Orpana H, Vachon J, Dykxhoorn J, McRae L, Jayaraman G. Monitoring positive mental health and its determinants in Canada: the development of the Positive Mental Health Surveillance Indicator Framework. Health promotion and chronic disease prevention in Canada: research, policy and practice. 2016 Jan;36(1):1.

2) Skinner R, Irvine B, Branchard B, Williams G, Pearson C, Kaur J, Yao X, Merklinger L, Lary T. A contextual analysis of the Suicide Surveillance Indicators. Health promotion and chronic disease prevention in Canada: research, policy and practice. 2017 Aug;37(8):257.

3) CCDSS Mental Illness Working Group, CCDSS Science Committee, CCDSS Technical Working Group. Mental Illness in Canada, 2015. Health Promotion and Chronic Disease Prevention in Canada: Research, Policy and Practice. 2015 Aug;35(6):95.

4) Pearson C, Janz T, Ali J. Health at a Glance. Mental and substance use disorders in Canada. Ontario: Statistics Canada, Ministry of Industry. 2013.

5) Special Advisory Committee on the Epidemic of Opioid Overdoses. National report: Opioid-related Harms in Canada Web-based Report. Ottawa: Public Health Agency of Canada; December 2019. https://health-infobase.canada.ca/substance-related-harms/opioids

